# Anesthetic Preconditioning as Endogenous Neuroprotection in Glaucoma

**DOI:** 10.3390/ijms19010237

**Published:** 2018-01-13

**Authors:** Tsung-Han Chou, Ganeswara Rao Musada, Giovanni Luca Romano, Elizabeth Bolton, Vittorio Porciatti

**Affiliations:** Bascom Palmer Eye Institute, University of Miami Miller School of Medicine, 1638 NW 10th Avenue, Miami, FL 33136, USA; tchou@med.miami.edu (T.-H.C.); gxm529@med.miami.edu (G.M.R.); glr56@med.miami.edu (G.L.R.); exb562@med.miami.edu (E.B.)

**Keywords:** glaucoma, mouse, retinal ganglion cells, lidocaine, neuroprotection

## Abstract

Blindness in glaucoma is the result of death of Retinal Ganglion Cells (RGCs) and their axons. RGC death is generally preceded by a stage of reversible dysfunction and structural remodeling. Current treatments aimed at reducing intraocular pressure (IOP) are ineffective or incompletely effective in management of the disease. IOP-independent neuroprotection or neuroprotection as adjuvant to IOP lowering in glaucoma remains a challenge as effective agents without side effects have not been identified yet. We show in DBA/2J mice with spontaneous IOP elevation and glaucoma that the lifespan of functional RGCs can be extended by preconditioning RGCs with retrobulbar lidocaine in one eye at four months of age that temporary blocks RGC axonal transport. The contralateral, PBS-injected eye served as control. Lidocaine-induced impairment of axonal transport to superior colliculi was assessed by intravitreal injection of cholera toxin B. Long-term (nine months) effect of lidocaine were assessed on RGC electrical responsiveness (PERG), IOP, expression of relevant protein (BDNF, TrkB, PSD95, GFAP, Synaptophysin, and GAPDH) and RGC density. While lidocaine treatment did not alter the age-related increase of IOP, TrkB expression was elevated, GFAP expression was decreased, RGC survival was improved by 35%, and PERG function was preserved. Results suggest that the lifespan of functional RGCs in mouse glaucoma can be extended by preconditioning RGCs in early stages of the disease using a minimally invasive treatment with retrobulbar lidocaine, a common ophthalmologic procedure. Lidocaine is inexpensive, safe and is approved by Food and Drug Administration (FDA) to be administered intravenously.

## 1. Introduction

Blindness in glaucoma is the result of death of Retinal Ganglion Cells (RGCs) and their axons [1]. RGC death is generally preceded by a stage of reversible dysfunction and structural remodeling [2]. Current treatments aimed at reducing intraocular pressure (IOP) are ineffective or incompletely effective in management of the disease [3]. IOP-independent neuroprotection or neuroprotection as adjuvant to IOP lowering in glaucoma remains a challenge as effective agents without side effects have not been identified yet [4]. The purpose of this study is to test the hypothesis that the lifespan of functional RGCs in mouse glaucoma can be extended by preconditioning RGCs in early stages of the disease using a minimally invasive treatment with retrobulbar lidocaine, a common ophthalmologic procedure. In vitro and in vivo studies have shown that lidocaine is neuroprotective against hypoxia and ischemia [5]. The primary action of retrobulbar lidocaine on the optic nerve is to inhibit transmembrane ionic fluxes [6] resulting in reduction of afferent electrical activity to thalamic targets of the visual pathway as well as inhibition of bi-directional axonal transport [7,8]. Axon transport plays a critical role in supplying materials for a variety of neuronal functions such as morphogenetic plasticity, synaptic transmission, and cell survival [9]. Previous results of our group in mice showed that retrobulbar lidocaine temporarily reduces RGC electrical responsiveness, which however rapidly recovers baseline values [10]. Chou and Porciatti [10] proposed that lidocaine-induced, temporary reduction of RGC electrical responsiveness is secondary to inhibition of axonal transport of target-derived factors that retrogradely sustain RGC function [11,12]. Here we show that short-term retrobulbar lidocaine administration in early stages of mouse glaucoma has a long-term effect on the expression of relevant retinal proteins and sparing of functional RGCs.

## 2. Results

### 2.1. Retrobulbar Lidocaine Impairs Axon Transport

**Figure 1:**
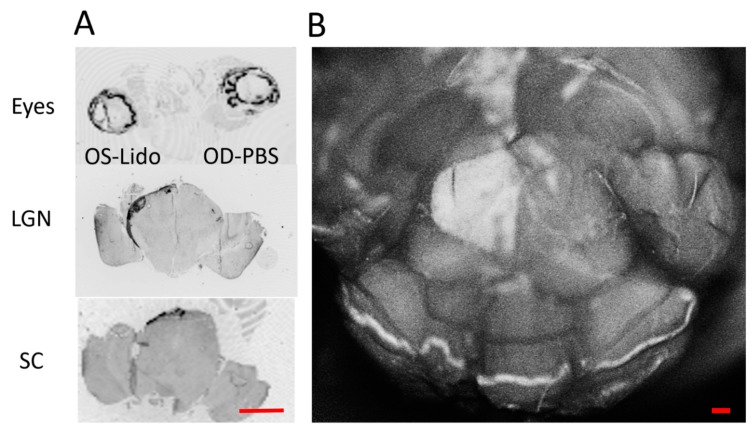
Retrobulbar lidocaine blocks axon transport. (**A**) Representative consecutive tissue sections from the eyes to the brain one day after retrobulbar lidocaine in the left eye, immediately followed by intravitreal injection of Alexa Fluor 488 Cholera Toxin B in both eyes. Fluorescence is evident in both eyes but it is absent in the lateral geniculate nucleus (LGN) and Superior Colliculi (SC) contralateral to the left eye. Fluorescence is present in the LGN and SC contralateral to the phosphate-buffered saline (PBS)-injected right eye; scale bar, 200 µm. (**B**) Confocal scanning laser ophthalmoscopy of the midbrain surface. The entire surface of the right SC receiving afferents from the lidocaine- injected right eye does not fluoresce, indicating impairment of axon transport in the lidocaine-treated eye; scale bar, 200 µm.

To confirm previous reports [7] that retrobulbar lidocaine impairs axonal transport, we intravitreally injected Cholera toxin b-subunit conjugated to AlexaFluor488 (CTB) in both eyes immediately after lidocaine retrobulbar injection in the left eye and retrobulbar phosphate-buffered saline (PBS) injection in the right eye. The next day, the entire brain was fixed with 4% paraformaldehyde in 0.5 M phosphate buffer pH 7.35 and dissected from the skull to expose the Superior Colliculi (SC). Confocal Scanning Laser images (Spec-CAM-11796-S3610) of the brain surface were taken to assess fluorescence of SC. As shown in Figure 1B, only the left SC (contralateral to the PBS-injected right eye) displayed marked fluorescence, whereas no fluorescence was appreciable in the right SC (contralateral to the lidocaine-injected eye). Then, consecutive tissue sections from the eyes to the brain were imaged with a fluorescent scanner (Typhoon, Amersham). Representative sections displayed in Figure 1A show that while CTB fluorescence was present in both eyes, fluorescence was only present in the outer shell of the lateral geniculate nucleus (LGN) receiving the PBS-injected contralateral axons; the inner core of the LGN receiving the lidocaine-injected ipsilateral axons did not fluoresce. In addition, only the SC contralateral to the PBS-injected eye displayed fluorescence. Altogether, results summarized in Figure 1 demonstrate that retrobulbar lidocaine inhibits axon transport to thalamic and midbrain targets.

### 2.2. Retrobulbar Lidocaine Reversibly Impairs RGC Function

**Figure 2:**
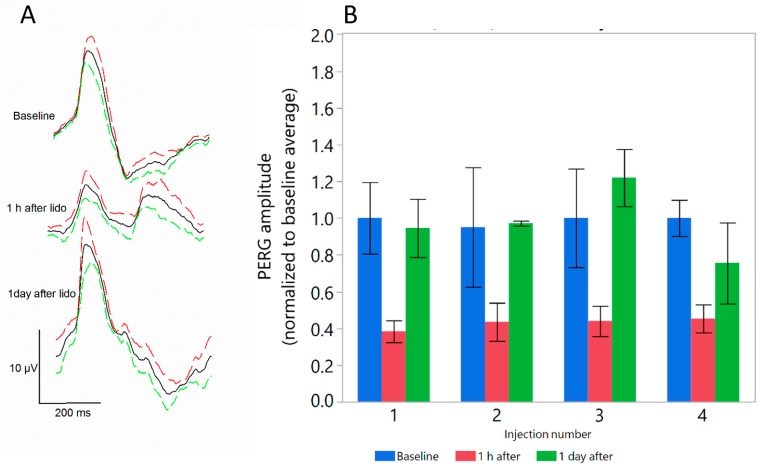
Retrobulbar lidocaine reversibly reduces Pattern Electroretinogram (PERG). (**A**) PERG waveforms in DBA/2J mice before and at different times after injection. Continuous black lines represent the grand-average waveform of all mice tested, and colored dashed lines represent the superior (red) and inferior (green) 95% confidence interval of the mean; (**B**) Peak-to-trough PERG amplitude decreases by about 60% one hour after lidocaine but recovers baseline values one day after injection. The effect is repeatable after four injections four days apart. Bars represent the mean ± SEM (*N* = 5).

Figure 2 shows that 1 h after retrobulbar lidocaine injection, RGC function measured with the Pattern Electroretinogram (PERG) [13] was reduced by more than 50%, however it fully recovered the next day. After PERG recovery, further lidocaine injections produced similar results. The temporary effect of lidocaine on PERG can be explained assuming impairment of retrograde signaling to the retina of factors that sustains RGC responsiveness [10]. Altogether, results summarized in Figure 2 indicate that the effect of lidocaine on PERG is repeatable, and that multiple lidocaine injections do not appear to be toxic to the retina as shown by unaltered PERG.

### 2.3. Lidocaine Treatment Does not Induce Long-Term Changes of IOP and RGC Function

**Figure 3:**
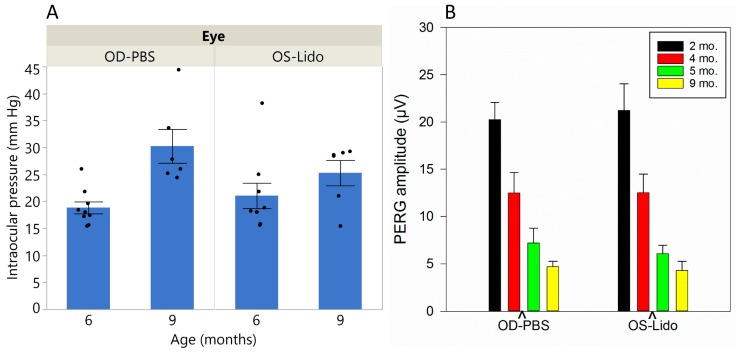
Intraocular pressure (IOP) and PERG amplitude as function of age in Lidocaine-treated and PBS-treated D2 mice. (**A**) IOP increases with age with no apparent differences between Lidocaine-treated and PBS-treated D2 mice. Bars represent the mean ± SEM (6 mo., *N* = 9; 9 mo., *N* = 6); black dots represent measurements in individual mice; (**B**) PERG amplitude declines with increasing age, with no apparent differences between Lidocaine-treated and PBS-treated D2 mice. Bars represent the mean ± SEM (2 mo., *N* = 7; 4 mo., *N* = 20; 5 mo., *N* = 9; 9 mo., *N* = 17).

In DBA/2J mice, IOP is known to progressively increase with age while PERG amplitude declines [14,15]. Results shown in Figure 3 confirm that this was also the case in our sample, with IOP increasing from about 20 mm Hg at six months of age to about 25 mm Hg at nine months of age (Figure 3A). However, repeated retrobulbar lidocaine injections in the left eye at four months of age did not cause significant IOP changes in the treated eye compared to the control eye (two-way ANOVA: effect of Age, *p* < 0.0018; interaction between age and treatment, *p* = 0.12). Figure 3B shows that PERG amplitude declined with increasing age. However, lidocaine treatment at four months of age did not induce further RGC dysfunction at five and nine months of age (2-way ANOVA: effect of age, *p* < 0.001, interaction between age and treatment, *p* = 0.6) Results suggests that lidocaine treatment at a young age does not have long-term toxic effects on RGC function as shown by a sensitive measure such as the PERG. 

### 2.4. Short-Term Lidocaine Treatment Induces Long-Term Changes of Protein Expression in DBA/2J Glaucoma

**Figure 4:**
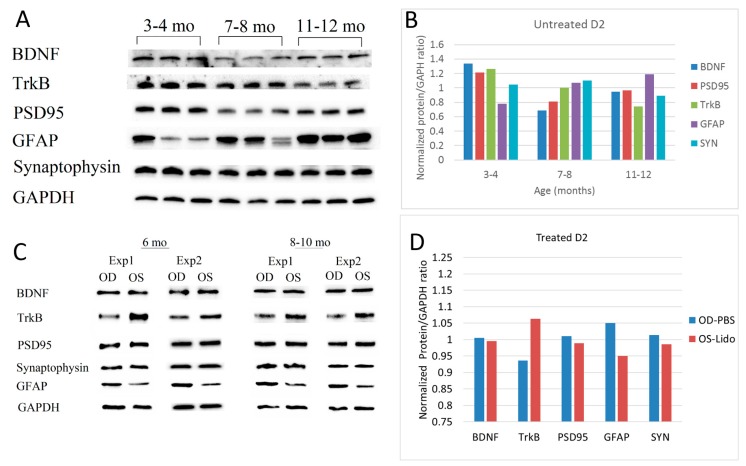
Age-related changes of relevant protein expression in untreated (**A**,**B**) and Lidocaine-treated (**C**,**D**) DBA/2J mice. (**A**) Western Blot images of Brain Derived Neurotrophic Factor (BDNF) (28 kDa), Tyrosine Receptor Kinase B (TrkB) (140 kDa), PSD95 (95 kDa), Synaptophysin (SYN) (38 kDa), Glial Fibrillary Acidic Protein (GFAP) (50 kDa) and Glyceraldehyde 3-phosphate Dehydrogenase GAPDH (37 kDa) proteins assessed in untreated mice of different ages (3 mice for age group, pooled retinas of both eyes); (**B**) Average protein expression (normalized protein/GAPDH ratio) as as function of age in untreated mice. Bars represent the average of 6 retinas for age group; (**C**) Representative Western Blot images of relevant retinal proteins in mice 6 and 8–10 old that received 4 retrobulbar injections of Lidocaine in the left eye and PBS in the right eye at 4 months of age; (**D**) Average protein expression in Lidocaine-treated (OS) and PBS-treated (OD) eyes. Bars represent the average ratios of 9 D2 mice of 6–10 months of age.

Previous analyses using microarrays, RT-PCR, and RNAseq, have shown marked changes of gene expression with increasing age in DBA/2J mice [15,16]. Western Blot analysis displayed in Figure 4A,B shows that in untreated DBA/2J mice, protein expression changed substantially with age. In particular, expression of TrkB progressively decreased with increasing age whereas expression of GFAP increased and expression of Synaptophysin was virtually invariant. Expression of BDNF and PSD95 tended to decrease in a nonlinear manner. However, when DBA/2J four month old received repeated retrobulbar Lidocaine injections, protein expression substantially was altered in the treated eye compared to the control eye 2–6 months later. Figure 4C,D shows that in the lidocaine treated eye, compared to the PBS control, the expression of TrkB was relatively increased while that of GFAP was relatively reduced, in countertendency with the natural history of age-related changes shown in Figure 4B. The expression of other proteins appeared unaltered.

### 2.5. Short-Term Lidocaine Treatment Results in Improved Long-Term Survival of Functional RGCs

**Figure 5:**
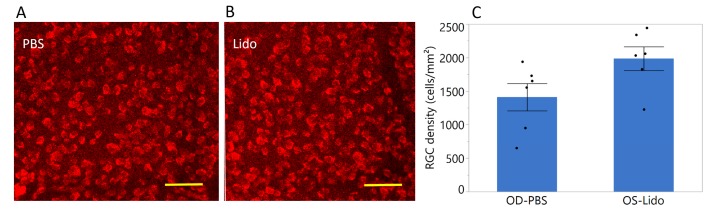
Retrobulbar lidocaine is neuroprotective in early DBA/2J glaucoma. (**A**,**B**) examples of confocal images of RBPMS-positive RGCs in whole-mounted retinas of one nine-month-old D2 that received four retrobulbar injections of lidocaine at four months of age in one eye (**B**) and an equal volume of PBS in the other eye (**A**), scale bar, 50 µm; (**C**) Mean RGC density in D2 mice nine mo. old treated with retrobulbar lidocaine in one eye and PBS in the contralateral eye at four months of age. RGC density is higher in lidocaine-treated eyes by 35% (*p* = 0.04). Error bars represent the SEM, *N* = 6. Black dots represent measurements in individual mice.

At 9 months of age, early manifest RGC death is normally present in D2 mice [17]. Figure 5 shows that RGC survival in nine-month-old D2 mice was significantly improved after short-term lidocaine treatment performed at four months of age. In Lidocaine-treated eyes, RGC density was higher by 35% compared to the PBS-treated eyes (*p* = 0.04, *N* = 6).

## 3. Discussion

Previous studies have shown that local anesthetic lidocaine is neuroprotective in in vitro and in vivo models of hypoxia and ischemia, as well as after spinal cord injury [5]. It has been suggested that reduction of neuroinflammation and inhibition of apoptosis contribute to neuroprotection. The present study investigated the potential role of lidocaine in the well-established DBA/2J mouse model of glaucoma, which has the hallmarks of human glaucoma including elevated IOP and chronic/progressive degeneration of retinal ganglion cells. Ischemia and neuroinflammation also may contribute to the disease [18,19,20].

The main result of the present study is that repeated retrobulbar lidocaine performed at four months of age—before the onset of IOP elevation and RGC dysfunction and death in DBA/2J glaucoma—resulted in long-term neuroprotection of functional RGCs. At nine months of age, when RGC death is normally present in about 30% of DBA/2J mice [17], lidocaine-treated eyes had RGC density significantly higher by ~35% on average compared to control, PBS-treated eye. The amplitude of the PERG, a sensitive measure of RGC function, was similar in the two eyes. This indicates that repeated lidocaine treatment at pre-glaucomatous ages was not toxic to RGCs, and that surviving RGCs were still functional. Intraocular lidocaine has been previously shown not to alter the ERG of rabbits [21].

One contributing factor to the neuroprotective effect of lidocaine might have been the upregulation of TrkB, which promotes cell survival [22,23] and is altered in experimental glaucoma [24]. This occurred in countertendency to progressive decline of TrkB expression with increasing age in untreated DBA/2J mice. Another contributing factor might have been the downregulation of GFAP, which indicates reduced astrocytic activation and inflammation [19,25,26], whereas in untreated DBA/2J mice there was a progressive increase of GFAP expression with increasing age. A third contributing factor might have been the marginally lower IOP in the lidocaine-treated eyes compared to the PBS-control eyes at nine months of age. Even though the IOP difference between the two eyes was not significant, a small difference at a high baseline level may have played a role in reducing the likelihood of cell death [27,28]. Intravenous lidocaine has been shown to attenuate the increase in intracranial pressure and IOP of patients receiving succinylcholine prior to intubation [29] and lower IOP in acute primary angle closure glaucoma [30]. Altogether, TrkB upregulation, GFAP downregulation, and IOP lowering may have played a synergistic role in the neuroprotective effect of lidocaine in DBA/2J glaucoma. Further investigation in a larger sample of mice including more advanced stages of the disease is needed to fully establish the neuroprotective effect of lidocaine and contributing factors.

Ischemic preconditioning is known to induce retinal tolerance to ischemia and optic nerve injury [31,32,33]. This study shows that the lifespan of functional RGCs in mouse glaucoma can be extended by preconditioning RGCs in early stages of the disease using a minimally invasive treatment with retrobulbar lidocaine, a common ophthalmologic procedure. Lidocaine is inexpensive, safe and is approved by FDA to be administered intravenously [5]. This study also provides further proof of principle that retinal ganglion cells have an intrinsic capacity to react to stress by changing expression of key proteins that sustain their survival [34,35,36]. Boosting endogenous neuroprotection with lidocaine may represent a novel therapeutic avenue in glaucoma.

## 4. Materials and Methods

### 4.1. Animals and Husbandry

All procedures were performed in compliance with the Association for Research in Vision and Ophthalmology (ARVO) statement for use of animals in ophthalmic and vision research. The experimental protocol was approved by the Animal Care and Use Committee of the University of Miami (IACUC Prot. 16-247, 6 July 2017). A total of 89 DBA/2J (D2), mice were used. The D2 mouse is a well-established genetic model of spontaneous glaucoma that has similarities with human pigmentary glaucoma [17,37]. By 5–6 months of age, D2 mice develop pigment dispersion in the iris that impairs aqueous humor outflow, resulting in moderate IOP elevation. By 5–6 months of age RGC function starts decreasing, and by 9–10 months the PERG amplitude is at noise level [14,15]. By 12 months of age D2 mice develop moderate or severe loss of RGC density [14,15,38]. All mice were maintained in a cyclic light environment (12 h light: 50 lux–12 h: dark) and fed with Grain Based Diet (Lab Diet: 500, Opti-diet, PMI Nutrition International, Inc., Brentwood, MO, USA). All procedures and testing were performed under anesthesia by means of intraperitoneal injections (0.5–0.7 mL/kg) of a mixture of ketamine (42.8 mg/mL) and xylazine (8.6 mg/mL). Retrobulbar injections of lidocaine (2 µL, 40 µg/µL) were performed in the left eye with a 23-gauge needle using a supraorbital approach. An equal volume of PBS was injected retrobulbar to the right eye as control. For experiments of long-term effects of lidocaine, repeated injections (*n* = 4 every 4 days) were done in mice four months old. Protein expression, RGC function and RGC density were quantified in the age range 6–10 months.

### 4.2. Pattern Electroretinogram (PERG) Recording

**Figure 6:**
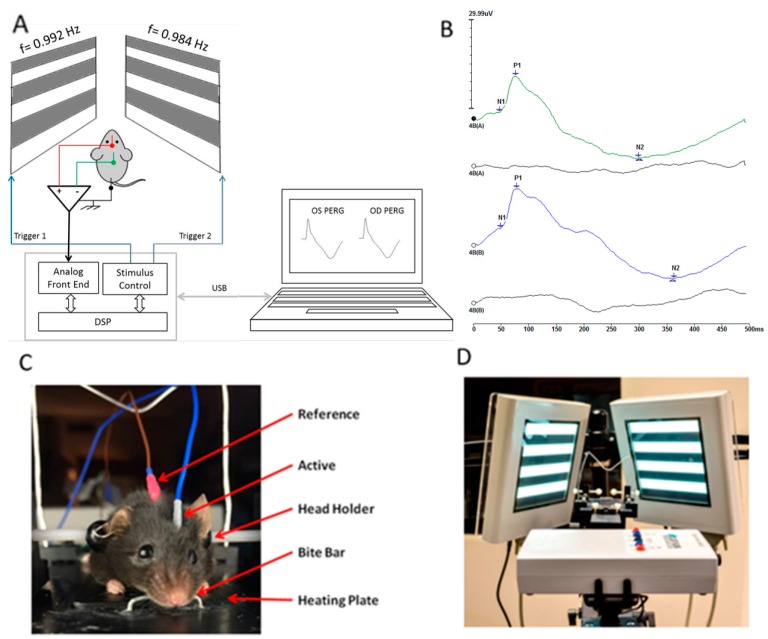
(**A**) Mouse PERG recording layout; (**B**) PERG waveforms simultaneously recorded from each eye using one common electrode in the snout; (**C**) Mouse with non-corneal subcutaneous needle electrodes resting on a feedback-controlled thermostatic plate; (**D**) Patterned visual stimuli generated on high luminance (800 cd/sqm) LED displays and presented at each eye independently with slightly different reversal frequency (right eye: 0.984 Hz; left eye: 0.992 Hz). Asynchronous averaging allows isolation of monocular PERGs.

As a sensitive measure of RGC function we used the Pattern Electroretinogram (PERG) [13,39], Figure 6. As previously described [39], anesthetized mice were gently restrained in a custom-made holder that allowed unobstructed vision. The body of the animal was kept at a constant temperature of 37.0 °C using a feedback-controlled heating pad (TCAT-2LV, Physitemp Instruments, Inc., Clifton, NJ, USA). The eyes of anesthetized mice were typically wide open and in a stable position, with optical axes pointing laterally and upwardly [25,26]. Pupils were natural and had a diameter smaller than 1 mm [40]; eyes were not refracted for the viewing distance since the mouse eye with natural pupil has a large depth of focus [41,42]. A small drop of balanced saline was topically applied as necessary to prevent corneal dryness. The PERG was recorded simultaneously from each eye using a common subcutaneous needle in the snout with a commercially available instrument (Jorvec Corp., Miami, FL, USA). Visual stimuli consisted of black-white horizontal bars generated on LED tablets and presented independently to each eye at 10 cm distance (56° vertical × 63° horizontal field; spatial frequency, 0.05 cycles/deg; 98% contrast; 800 cd/sqm mean luminance; right-eye reversal, 0.992 Hz; left-eye reversal, 0.984 Hz). Electrical signals recorded from the common snout electrode were averaged (>1110 epochs), and PERG responses from each eye isolated by averaging at stimulus-specific synchrony [39]. Examples of normal PERG waveforms are shown in Figure 1. PERG amplitude was defined as amplitude difference from the major positive peak (P1) to the major negative trough (N2). PERG latency was defined as the time lag from zero (onset of reversal) to the peak of the P1 wave. 

### 4.3. Western Blots

Expression of relevant retinal neurotrophic expression (BDNF, TrkB), synaptic markers (PSD95, synaptophysin) and glial markers (GFAP) was performed with Western Blot. Eyes were harvested, and retinae dissected from the pigment epithelium. Retinae from each eye were pooled separately and homogenized in RIPA buffer containing 1XHALT protease inhibitor cocktail (Pierce, Rockford, IL, USA). The protein concentrations of retinal homogenates were estimated using DC protein assay (Bio-Rad, Hercules, CA, USA). Equal concentration of the total protein samples (40 µg) were added to the Laemmli buffer, boiled at 95 °C for 5 min ad further electrophoresed on 4–20% Mini-Protean TGX precast gels (Bio-Rad). The separated proteins were transferred onto a 0.2 μm polyvinylidene difluoride membranes (PVDF, Bio-Rad). Membranes were blocked in 5% milk in 1× Tris-buffered saline + Tween (TBST) for 1 h at room temperature. The blocked membranes were incubated with primary antibody for overnight at 4 °C. Membranes were washed three times for 15 min, and then incubated with secondary antibody for 60 min at room temperature. For each sample, three gel runs were performed. To normalize for protein loading, membranes were stripped and then reprobed with constitutively expressing housekeeping gene (GAPDH). Primary were: BDNF (Novus, Littleton, CO, USA) NBP2-36705, 2 µg/mL; TrkB (Cell Signaling Technology, Danvers, MA, USA) #4603, 1:1000; PSD95 (Abcam, Cambridge, MA, USA) ab76115, 1:1000; GFAP (Cell Signaling Technology #12389, 1:1000), Synaptophysin (Cell Signaling Technology, #5461, 1:1000) and GAPDH (Cell Signaling Technology, #8884, 1:1000). Membranes were washed three times for 15 min and developed with enhanced chemiluminescence (ECL; Bio-Rad) solution. The developed bands were quantified using ImageJ software for gel densitometry (provided in the public domain by the National Institutes of Health, available online: http://rsbweb.nih.gov/ij/download.html). The relative density of protein bands was normalized to the levels of GAPDH (protein/GAPDH) within each sample by dividing the percent value for each sample by the percent value for the GAPDH standard.

### 4.4. RGC Density

RGC density was assessed in whole-mounted retinas immunostained with RBPMS primary antibody (PhosphoSolutions, Aurora, CO, USA) 1832-RBPMS, 1:500 plus Alexa 594 secondary antibody (Abcam, ab150188, 1:500) that stains all RGCs [43], and manually quantified with confocal microscopy images.
